# Unravelling the Link Between Hidradenitis Suppurativa and Inflammatory Bowel Disease: A Literature Review

**DOI:** 10.3390/biomedicines13081833

**Published:** 2025-07-27

**Authors:** Konstantinos Mpakogiannis, Fotios S. Fousekis, Emmanouil Karampinis, Eleftheria Mastoridou, Georgios Gaitanis, Konstantinos H. Katsanos

**Affiliations:** 1 Division of Gastroenterology, Department of Internal Medicine, Faculty of Medicine, School of Health Sciences, University of Ioannina, 45110 Ioannina, Greece; kostismpakogiannis@gmail.com (K.M.); fotisfous@gmail.com (F.S.F.); e.mastoridou@uoi.gr (E.M.); 2 Second Dermatology Department, School of Health Sciences, Aristotle University of Thessaloniki, 54124 Thessaloniki, Greece; emankarampinis@gmail.com; 3 Department of Dermatology, University General Hospital of Larissa, Faculty of Medicine, School of Health Sciences, University of Thessaly, 41110 Larissa, Greece; 4 Department of Dermatology and Venereology, Medical School, University of Ioannina, 45110 Ioannina, Greece; ggaitan@uoi.gr

**Keywords:** hidradenitis suppurativa, Crohn’s disease, ulcerative colitis, inflammatory bowel disease, immunopathogenesis

## Abstract

Hidradenitis suppurativa (HS) and inflammatory bowel disease (IBD), including Crohn’s disease (CD) and ulcerative colitis (UC), are chronic, immune-mediated conditions with significant impact on quality of life. Emerging evidence reveals a notable epidemiological and pathogenic overlap between HS and IBD, particularly CD. Although a bidirectional association between HS and IBD has been well documented, current evidence supports a causal effect of IBD on the development of HS, while a causal relationship in the opposite direction has yet to be established. The present review explores the important association between these immune-mediated conditions and further highlights shared risk factors, genetic predispositions and immunopathogenic mechanisms, such as dysbiosis and cytokine dysregulation, involved in both HS and IBD. Diagnostic challenges, especially in differentiating perianal HS from perianal CD, are also discussed. The coexistence of HS and IBD impacts disease severity, treatment response, and overall management strategies. Shared therapeutic approaches, such as TNF-α inhibitors and JAK inhibitors, are considered promising options for effectively managing patients affected by both conditions. Nevertheless, deeper understanding of the gut–skin axis that will offer potential for more precise interventions in patients with simultaneous HS and IBD is considered imperative.

## 1. Introduction

Hidradenitis suppurativa (HS) is a chronic, recurrent skin condition diagnosed based on three criteria: (1) the presence of typical lesions such as deep-seated painful nodules, abscesses, draining sinuses, and scarring, (2) involvement of typical predilection areas including the axillae, groin, perineum, inframammary and intermammary folds, or buttocks, and (3) a history of chronicity and/or recurrence of these lesions [1,2]. Its pathophysiology is still being elucidated but is believed to involve follicular occlusion, dysregulated innate immunity, and aberrant inflammatory responses [3]. HS prevalence is 0.1% in the USA and ≥1% in Europe. It is also more common in Western women, with the female-to-male ratio being approximately 3:1, while the same ratio is 1:2 in South Korean patients [4]. IBD, including Crohn’s disease (CD) and ulcerative colitis (UC), is a group of immune-mediated conditions involving inflammation of the gastrointestinal tract, that result in symptoms such as abdominal pain, diarrhea, and rectal bleeding [5]. IBD was initially reported in Western nations, but its incidence is now rapidly increasing in newly industrialized regions like Asia, South America, eastern Europe, and Africa, with similar rates observed in both males and females worldwide [6]. Both HS and IBD are linked to mental health challenges, including depression and anxiety and significantly impair quality of life and work productivity, with the impact being greater in HS than in IBD [1,3,7,8,9]. HS, characterized by recurrent painful abscesses, and pruritus, leads to chronic physical pain, emotional distress, and social isolation, often resulting in low self-esteem, sleep disturbances, and poor mental health, while IBD primarily affects physical health through gastrointestinal symptoms and disrupts daily life with issues like pain, fatigue, and the constant fear of flare-ups [1,3,7,8,9]. Chronic inflammatory diseases such as HS and IBD present complex diagnostic and therapeutic challenges, not only due to their systemic manifestations but also because of their frequent co-occurrence and increasing recognition of shared epidemiological and pathogenic features [10,11]. Multiple cohort studies have reported that patients with IBD are significantly more likely to develop HS, and vice versa, establishing HS as an IBD-associated cutaneous manifestation [12,13,14]. This overlap has generated scientific interest regarding whether the co-occurrence of these diseases is attributable to shared risk factors and immune-mediated pathways, or whether one condition directly contributes to the onset or exacerbation of the other [15]. Understanding the relationship between HS and IBD holds significant clinical implications, as it may facilitate earlier diagnosis, inform integrated treatment strategies, and ultimately improve outcomes for patients affected by these frequently coexisting inflammatory disorders. This article examines the current evidence supporting a potential association between HS and IBD, elucidates their shared pathogenetic mechanisms, and outlines common therapeutic approaches.

## 2. Materials and Methods

A literature review was conducted using the PubMed and Embase databases up to 20 March 2025, aiming to identify relevant publications addressing the potential association between IBD and HS. The following search strings were used: “Inflammatory bowel disease”, “IBD”, “Crohn’s disease”, “Ulcerative colitis”, “Hidradenitis suppurativa”, “acne inversa”. All selected articles were further screened for additional relevant articles through their reference lists. Also, there were no language restrictions in the selection of the desirable articles.

## 3. Results and Discussion

### 3.1. The Potential Bidirectional Association Between HS and IBD

According to the current literature, HS is a relatively uncommon immune-mediated condition that can occasionally coexist with CD or UC [16,17,18]. A study conducted by Van Der Zee et al. was the first to investigate the prevalence of HS among patients with IBD [19]. In a cohort of 158 patients with IBD, 16% reported a history of recurrent painful boils in the axillary and/or inguinal regions, consistent with HS, with a prevalence of 17% among those with CD and 14% among those with UC [19]. These findings suggested that HS can occur in CD or UC [19]. A population-based cohort study demonstrated that patients with IBD have an almost ninefold increased risk of developing HS compared to the general population (incidence rate ratio: 8.9; 95% CI, 3.6–17.5) [20]. Over a median follow-up of 19.8 years, HS developed in 1.18% of 679 IBD patients, with a strong female predominance [20]. A nationwide cohort study utilizing data from the Taiwan National Health Insurance Research Database demonstrated that HS is not only more prevalent among patients with IBD but also tends to develop subsequent to the diagnosis of IBD, with a hazard ratio (HR) of 2.48 (95% CI, 1.03–5.97) [14]. This suggests a possible temporal and progressive relationship between gut and skin inflammation [14]. A pooled analysis of four studies further substantiated the epidemiological association between these conditions, reporting an overall prevalence of HS among patients with IBD of 12.8% (95% CI: 11.7–13.9%) [10]. When stratified by IBD subtype, HS was determined in 17.3% of CD patients and 8.5% of those with UC, reinforcing a stronger association with CD [10]. The prevalence of IBD among HS patients consistently exceeds that of the general population, considering HS as a potential risk factor for IBD development [21,22,23]. In a large Swedish registry-based study involving 13,538 patients with HS, the prevalence of inflammatory bowel disease was 3%, markedly higher than estimates observed in the general population [21]. Similarly, a multicenter cross-sectional study involving 1076 HS patients found a 3.3% prevalence of IBD (2.5% for CD, 0.8% for UC), markedly higher than the general northern European prevalence of 0.41–0.74% [22]. A large Danish national registry study further substantiated these findings, reporting that HS patients had significantly increased odds of co-occurring CD (OR 2.04) and UC (OR 1.75) and were also at elevated risk for developing new-onset IBD (HR 2.19 for CD, HR 1.63 for UC) [13]. Major meta-analyses further confirmed the above findings [12,24]. In particular, Phan et al. identified a pooled odds ratio of 2.12 for IBD in HS cohorts, with stronger associations noted for CD (OR 2.25) compared to UC (OR 1.56) [24]. Similar findings were reported by Chen et al., who emphasized the importance of gastrointestinal consultation for HS patients presenting with gastrointestinal symptoms [12]. Fecal calprotectin may serve as a useful screening tool for detecting IBD in patients with HS [25,26]. In an Israeli cohort of 3207 HS patients, HS was significantly associated with CD (OR 2.03) but not UC, even after multivariate adjustment [27]. A large U.S.-based cohort study demonstrated that patients with HS have more than twice the risk of developing UC (HR 2.30) and CD (HR 2.70) compared to matched controls without chronic inflammatory skin conditions [28]. The above, may indicate a potential association between HS and IBD [10,12,13,14,16,17,19,20,21,22,23,24,27,28]. However, it is important to highlight that the first study investigating the causal association between IBD and HS, utilizing a randomized Mendelian clinical trial design, which is well regarded for establishing causality and minimizing confounding factors, was conducted by Bao et al. [29,30]. The study demonstrated that IBD and its subtypes may have a causal effect on the development of HS, whereas no causal effect was identified in the reverse direction [29]. These findings underscore the critical need for further research to elucidate gut–skin axis interactions and to explore the potential causal relationship between IBD and HS [29]. Important original studies investigating the potential bidirectional association between HS and IBD are summarized in Table 1.

### 3.2. The Notable Association and Differential Diagnosis of CD and HS

**Figure 1:**
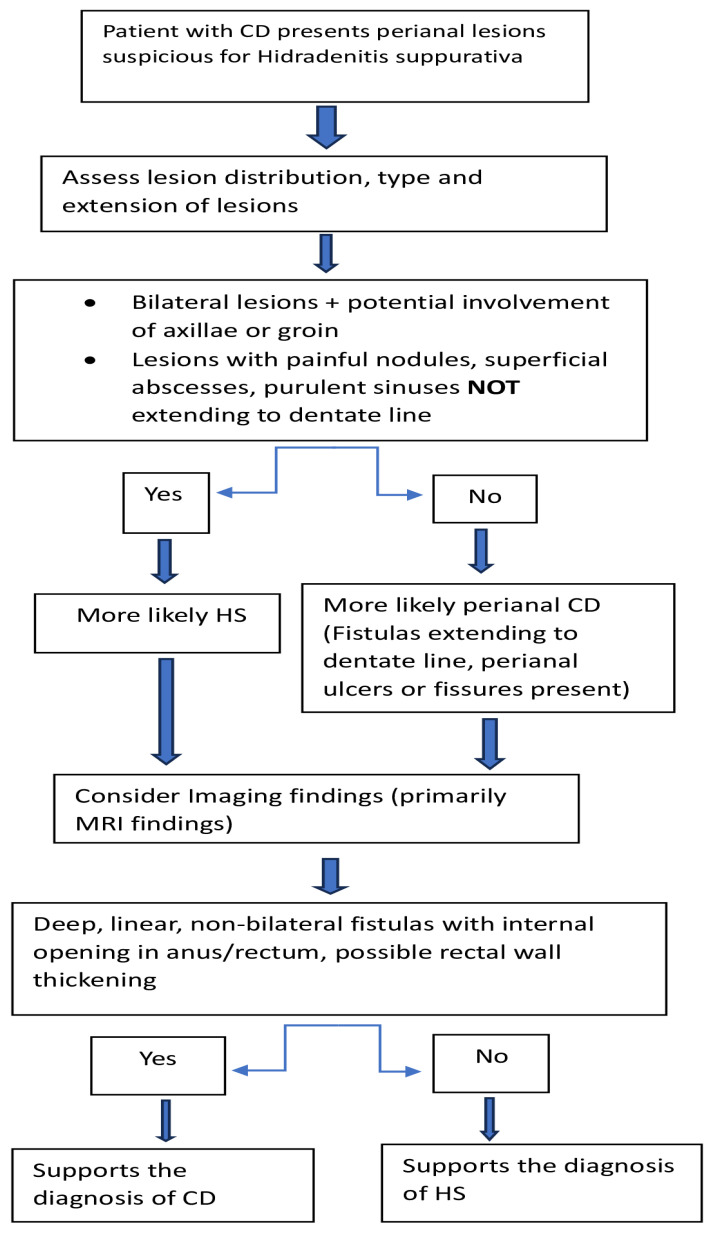
Suggested algorithm for diagnosis of HS in patients with CD and perianal lesions.

Regarding the subtype of IBD most strongly associated with HS, CD demonstrates a higher prevalence compared to UC, suggesting shared pathogenetic mechanisms underlying both conditions [10,13,15,18,22,24,28,31]. Several cases have supported the significant relationship between CD and HS [32,33,34,35,36]. The prevalence of HS among patients with CD ranges between 12% to 18% [37]. In contrast, approximately 3% of individuals with HS are also diagnosed with CD [22,37]. CD predominantly precedes HS, suggesting that HS may act as a cutaneous extraintestinal manifestation of CD [20,33,38,39]. Patients with coexistence of CD and HS are more likely to exhibit a higher severity of HS (Grade 2,3 according to Hurley staging for HS, Table 2), earlier onset of CD, as well as a more severe CD phenotype, including greater colonic or ileocolonic involvement, higher rates of perianal disease, and an increased requirement for surgical intervention, accompanied by a correspondingly higher probability of necessitating a permanent stoma [18,37,38,40,41,42,43]. Kamal et al. conducted a retrospective analysis of 15 patients with both CD and HS, reporting that 47% had colonic CD, 53% had ileocolonic involvement, and 67% presented with perianal disease, with no cases of isolated ileal disease [41]. HS lesions were most commonly located in the perianal/perineal (73%), axillary (53%), and inguinal (47%) regions, and the majority of patients (93%) had Hurley stage II or III [41]. Supporting these findings, another retrospective case–control study reported that 80% of patients with concurrent CD and HS had Hurley stage II or III disease [38]. These patients also exhibited more active CD (56% vs. 40%, *p* < 0.001) and significantly higher risk of requiring a permanent stoma (16.8% vs. 2.5%, *p* = 0.002) [38]. Lukach et al., similarly to Kamal et al., identified the inguinal, perianal, and axillary regions as the most frequently affected sites in patients with both HS and IBD [40]. In their case–control study, patients with both CD and HS were significantly more likely to have ileocolonic involvement (OR 8.31, 95% CI 2.90–23.80) and perianal disease (OR 2.85, 95% CI 1.19–6.81) compared to those with CD alone (*p* < 0.01 for both) [40]. In the study published by Tandon et al., those with HS and CD were additionally more likely to present active perianal disease (OR 21.1, 95% CI 6.2 to 71.9, *p* < 0.005) [42]. Differentiating perianal CD from perianal HS remains challenging, as HS can coexist with CD vice versa, and also perianal CD and perianal HS can have similar clinical manifestations, such as abscesses or sinus tracts accompanied by perianal pain, itching, redness, bleeding, and increased purulent secretion [44]. HS is an autoinflammatory skin disorder that affects the apocrine glands of hair follicles and tends to localize superficially without extending to the dentate line, while CD is a transmural gastrointestinal inflammatory disease that commonly presents deep, penetrating fistulas possibly involving the dental line and perianal structures [45,46]. Imaging studies, primarily MRI and U/S are critical: HS-related tracts are more superficial and often bilateral, whereas CD fistulas typically show deep, unidirectional tracts with internal opening in the anus or low rectum [47]. Monnier et al. found that a combination of imaging features, specifically posterior lesion involvement, bilateral distribution, and absence of rectal wall thickening, had an 100% specificity (95% CI: 92.3–100) for diagnosing HS [48]. Furthermore, patients with perianal CD often exhibit perianal fissures and ulcers, gastrointestinal symptoms (pain, rectal bleeding, diarrhea), as well as lesions in the buttocks and perineum, while those with perianal HS present additional lesions in the axillae and groin with simultaneous absence of gastrointestinal symptoms and perianal fissures and ulcers [15,49]. Laboratory testing of anemia profiles and emerging inflammatory markers, such as the monocyte-to-lymphocyte ratio (MLR) and platelet-to-lymphocyte ratio (PLR), showed significant elevation in the IBD group, further supporting differentiation between conditions, with CD exhibiting higher levels of systemic inflammation and anemia [50]. Histologically, foreign body granulomas, typically observed near occluded follicles, have been considered indicative of HS, while deeper epithelioid granulomas are more commonly associated with CD [51,52,53]. The recent literature highlights substantial histological overlap between perianal HS and perianal CD, indicating that the presence of granulomas and lymphoid follicles are insufficient to reliably differentiate between the two conditions [15,54]. Important original studies investigating the characteristics of patients with both HS and IBD are summarized in Table 3, while the major differences between CD and HS are summarized in Table 4. A suggested diagnostic algorithm for diagnosis of HS in CD patients with perianal lesions is illustrated in Figure 1.

### 3.3. Potential Epidemiological Factors Associated with the Co-Occurrence of HS and IBD

Obesity, smoking, sex, age, and race have been discussed as potential factors associated with the development and co-occurrence of HS and IBD [10,44,55]. According to studies by Yadav et al. and Lukach et al., obesity is more prevalent among patients with coexisting IBD and HS compared to those with IBD alone [20,40]. Specifically, in the prospective study conducted by Yadav et al., the majority of IBD patients who developed HS (6 out of 8) were obese [20]. Furthermore, in the case–control study by Lukach et al., patients with both HS and IBD were nearly 11 times more likely to be obese (*p* < 0.01) compared to controls with IBD alone [40]. Conversely, in the study conducted by Garg et al., the prevalence of CD among HS patients was highest among non-obese individuals (2.8%) compared to obese patients (*p* < 0.001), while Deckers et al. similarly found a lower obesity rate in HS-IBD patients (13.9%) compared to HS-only patients (31.2%; *p* = 0.04) [22,56]. In addition to this, another case–control study suggested that obesity was significantly less frequent in patients with both HS and IBD (Cases) compared to those with HS but no IBD (Controls) (4% vs. 25%, *p*-value = 0.02) [57]. Therefore, obesity is a potential risk factor for HS development in patients with pre-existing IBD, but it may be less prominent when IBD follows the appearance of HS [20,22,40,56,57]. This aligns with a recent meta-analysis highlighting that obesity is not consistently linked to an increased risk of developing IBD [58]. Several studies have explored the relationship between smoking, HS, and IBD, particularly CD [22,40,42,56]. Lukach et al. found that current smoking was nearly six times more common in patients with both HS and IBD compared with patients presenting only IBD (*p* < 0.01) [40]. Similarly, Tandon et al. showed that current and past smoking is a potential risk factor for developing HS in IBD patients [42]. However, Garg et al. added another layer of CD occurrence in HS patients [56]. They found that 2.3% of HS patients who smoked had CD, indicating a higher prevalence compared to the general population [56]. Nevertheless, in the same study, the strongest association between HS and CD was observed in nonsmokers [56]. Thus, even in the absence of smoking, the existence of HS significantly increases the risk of developing CD [56]. This is further supported by the results of a multicenter study describing no significant difference in smoking status between patients with both HS and IBD, and those with HS alone [22]. While smoking increases the risk of HS in IBD patients, HS itself may be a strong independent risk factor for CD occurrence, even without the influence of smoking [22,56]. The bidirectional association between HS and IBD is further influenced by several demographic factors, notably sex and race [20,22,42,55,56]. In IBD patients, those who develop HS are generally younger and more frequently African-American females [20,42,55]. Conversely, in HS patients, a stronger association with IBD development has been described in white males, aged 45–64 according to the large sample study conducted by Garg et al. [56]. The study compiled by Deckers et al. also indicated that there was no statistically significant difference regarding the gender between patients with IBD and HS and patients exhibiting only HS [22]. From the aforementioned evidence, it is clear that while smoking, obesity, female sex, black race, and young age significantly increase the risk of HS in patients with IBD, HS itself may be the strongest independent risk factor associated with the development of IBD, particularly CD, in patients with pre-existing HS. This further reinforces the perspective that IBD may contribute causally to the development of HS, with HS potentially serving as a secondary cutaneous manifestation of underlying IBD-related inflammation, even in the uncommon instances where HS precedes the onset of IBD [15,29,59].

### 3.4. Genetic and Immunopathogenic Overlap Between IBD and HS

The genetic and immune link between HS and IBD is an area of growing interest as research increasingly supports the presence of overlapping molecular pathways and hereditary susceptibilities [10,29,60,61]. The above is additionally reinforced by the fact that HS and IBD share a multitude of common comorbidities [62,63,64,65,66,67]. NOD2 gene variations impair bacterial recognition and autophagic clearance, leading to excessive immune activation and CD development [68,69]. While NOD2 mutations are clearly established in CD occurrence, it has not been directly associated with classic HS [31]. However, NOD2 mutations have been determined in rare cases of PASH syndrome (pyoderma gangrenosum, acne, and HS) with concurrent gut inflammation [70,71]. Bridging the genetic gap between HS and IBD, Janse et al. identified three genes with altered expression in patients with simultaneous HS and IBD: *SULT1B1* and *SULT1E1* genes, which are associated with increased risk of coexisting IBD and HS, and the *ELOVL7* gene, which appears to be protective in the same context [60]. *SULT1E1*, which encodes an estrogen sulfotransferase, is also expressed in adipose tissue and has been shown to be co-expressed with TNF-α, a key proinflammatory cytokine in HS and IBD occurrence [10,72,73]. Gower-Rousseau et al. reported three cases of HS occurring in two first-degree relatives of individuals with CD, underlining the potential shared genetic susceptibility between the two conditions [74]. Microbiota alterations and intestinal dysbiosis emerge also as an important contributor in HS and IBD co-existence [31,75,76,77]. According to Eppinga et al., patients with concomitant HS and IBD exhibited a greater increase in *Escherichia coli* abundance as well as a more pronounced decrease in *Faecalibacterium prausnitzii* compared to patients with IBD alone [77]. In contrast, patients with a history of HS without IBD showed no significant differences in the abundance of *F. prausnitzii* or *E. coli* [77]. Moreover, in IBD, a reduction in *F. prausnitzii*, a bacterium with protective and anti-inflammatory properties, alongside an increase in adherent-invasive *E. coli*, which promotes intestinal inflammation, has been well documented [31,78]. While intestinal dysbiosis is observed in patients with simultaneous HS and IBD, it remains unclear whether these microbial alterations are a cause of IBD or a consequence of the chronic intestinal inflammation [31]. Interestingly, HS may involve an exaggerated immune response to normally harmless skin microbes, similarly to how CD patients respond abnormally to gut microbiota [44]. TLRs (Toll-like receptors) and inflammasomes can also be activated as a response to skin and gut microbial components, promoting inflammation in both skin and gut [31]. Although direct evidence on skin microbiota changes in HS is limited, emerging data suggest that microbial imbalances in the gut potentially influence skin inflammation, supporting the concept of a gut–skin axis [79,80]. Activation of the aryl hydrocarbon receptor by tobacco smoke possibly also serves as a shared molecular trigger in CD, with smoking contributing also to microbiota alterations, TNF-a secretion, neutrophil chemotaxis, and follicular obstruction [31,81,82]. Overlapping cytokine-mediated immune dysregulation further participates in the bidirectional association between HS and IBD [29]. IL-1, IL-6, IL-12, IL-17, IL-23, and TNF-a that are secreted by innate immune cells (mainly macrophages, dendritic cells) as well as by adaptive T-helper cells (particularly Th17/Th1), are critical cytokines linking HS and IBD [83,84,85]. Giudici et al. has also underlined the existence of numerous CD161+ T-lymphocytes in CD fistulas and HS lesions suggesting that these cells may play a pathogenic role in CD and HS co-occurrence [86]. IL-1, a key interleukin contributes to the inflammation process in both the skin and intestine leading to neutrophilic inflammation [87]. IL-17 has a crucial role in neutrophil recruitment as well as epithelial inflammation [84,88]. IL-12 and IL-23, secreted by macrophages, drive Th17/Th1 cell differentiation [84]. IL-6 further upholds chronic inflammation and Th17 differentiation [15]. It should be mentioned that IL-10, an anti-inflammatory cytokine, is possibly reduced in both diseases [89,90]. IL-36 and IL-38 are recently recognized interleukins of growing scientific interest, increasingly implicated in the pathogenesis of autoinflammatory diseases such as HS and IBD [91,92,93]. Interestingly, elevated STAT1 signaling involved in the JAK/STAT pathway is detected in lamina propria T-cells of CD patients, but not in those with UC, while increased STAT1 mRNA expression also characterizes the keratinocyte gene expression profile in HS [94,95]. The above finding supports the role of STAT1 as an additional potential molecular link between CD and HS [94,95]. Genetic and immunopathogenic factors contributing to overlap between HS and IBD are illustrated in Table 5.

### 3.5. Common Therapeutic Options for IBD and HS

**Figure 2:**
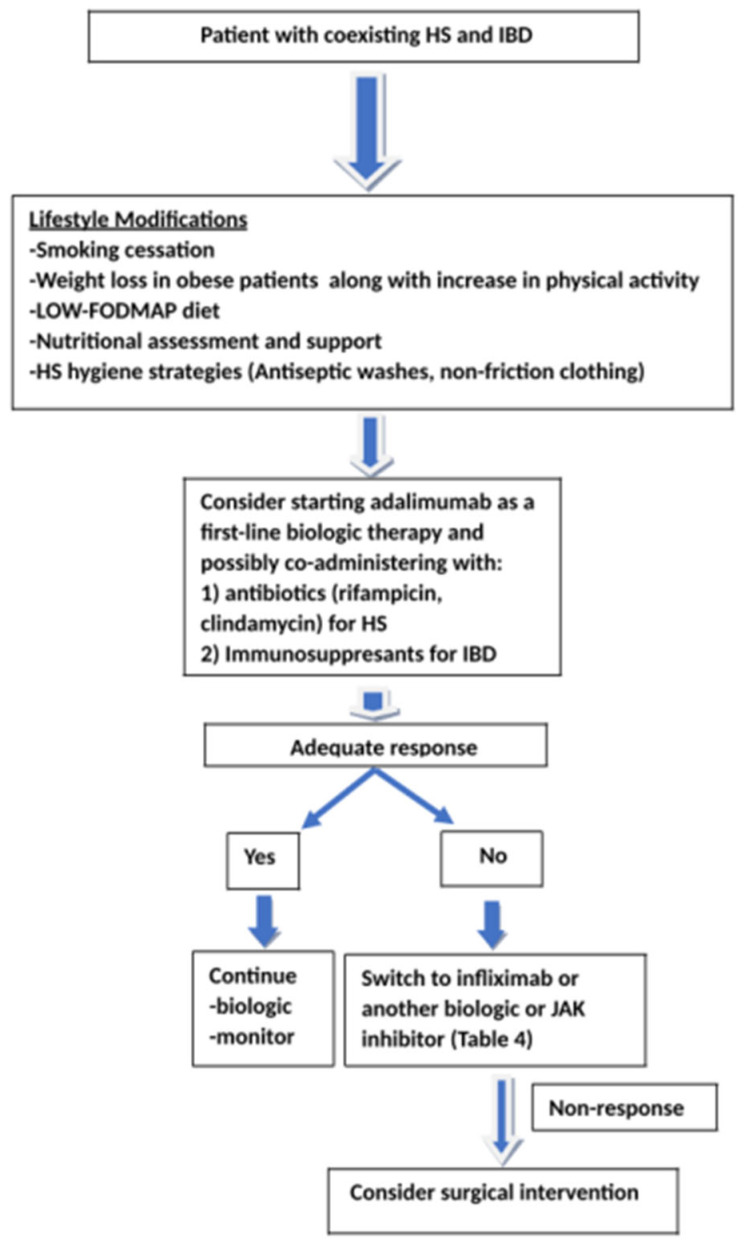
Potential treatment algorithm for patients with concurrent HS and IBD.

As already mentioned, despite affecting different organ systems, with IBD primarily targeting the gastrointestinal tract and HS affecting the skin and hair follicles, both conditions share overlapping pathogenic pathways [10,44]. This has led to the exploration of similar therapeutic strategies in their management that should be tailored to each patient, focusing on both controlling the underlying IBD and addressing HS [44,96]. It is important to emphasize that systemic antibiotics generally have limited efficacy in treating patients with HS and IBD [44]. However, antibiotics such as tetracyclines and clindamycin–rifampicin combinations may be effective for managing HS in such patients [44]. Immunomodulators also play a crucial role in regulating the immune response and maintaining remission in IBD [44]. Both HS and IBD may further benefit from biologic therapies, especially anti-TNF agents, which have shown efficacy in controlling both cutaneous and intestinal manifestations [10,44]. It should be noted that the coexistence of IBD and HS is linked to a greater need for anti-TNF therapy, more frequent anti-TNF dose escalation, and an increased likelihood of surgical resection compared to patients with either condition alone [20,38,41,60]. Adalimumab is the only FDA-approved anti-TNF biologic agent for the treatment of HS, also offering therapeutic benefits for patients with coexisting HS and IBD [10,97,98,99,100,101,102]. Infliximab, another anti-TNF biologic, is used off-label for the treatment of HS and has demonstrated both efficacy and safety in patients with coexisting IBD [103,104,105,106,107,108,109,110]. It is important to emphasize that there have been reported cases of patients with IBD treated with biologic agents, particularly anti-TNF therapies, who developed HS, perhaps as a paradoxical adverse effect, often necessitating discontinuation and a subsequent change to their biologic treatment [111,112,113,114,115,116,117,118,119]. In patients with HS and IBD who are refractory to anti-TNF therapy, alternative off-label options, such as an IL-12/IL-23 antagonist (ustekinumab) and JAK inhibitors (tofacitinib, upadacitinib), and IL-23 selective inhibitors (guselkumab), should be considered according to published cases [101,120,121,122,123,124,125]. Given their proven efficacy in IBD, tofacitinib and upadacitinib are also currently being evaluated in ongoing trials for their potential role in the treatment of HS [126,127]. Secukinumab and bimekizumab, known as IL-17 inhibitors, although effective for HS, have been associated with exacerbation of IBD and, therefore, should be avoided in patients with underlying IBD [88,128]. Furthermore, in cases where pharmacological therapies fail to control disease activity in patients with both HS and CD, surgical intervention may be required to achieve adequate disease control [44,129]. Other future therapeutic options for managing the often-coexisting conditions of HS and IBD, include high-dose vitamin B_12_ supplementation and adherence to a strict low-FODMAP diet, however further trials regarding these interventions are considered imperative [130,131]. It is also essential to emphasize that probiotics can play a pivotal role in restoring a healthy skin microbiome by promoting beneficial bacteria such as *Cutibacterium* spp., *Corynebacterium*, and *Staphylococcus*, which are often reduced in HS patients [80]. Furthermore, probiotics have the ability to modulate gut microbiota, inflammation, and oxidative stress, key factors that significantly influence overall skin health [80]. While the potential of probiotics in treating both HS and IBD is promising, further research is necessary to pinpoint the specific strains that may offer the most benefit [80,132]. Table 6 provides a summary of major therapeutic options (biologics and JAK inhibitors) targeting both HS and IBD, while Figure 2 illustrates a proposed treatment algorithm for patients with concurrent HS and IBD, based on current literature evidence.

## 4. Conclusions

The association between HS and IBD, primarily CD, highlights how distinct inflammatory conditions can share underlying immunopathogenic mechanisms. Epidemiological studies consistently demonstrate a potential bidirectional association, though current evidence from Mendelian randomization studies supports a causal effect of IBD on HS development, while the reverse has not been yet established. This relationship has important clinical implications. Diagnosing coexisting HS and IBD and also differentiating perianal HS from perianal CD remain challenging due to overlapping clinical features. For patients with HS who present with gastrointestinal symptoms, early screening for IBD may lead to improved clinical outcomes. Therapeutically, the overlap between HS and IBD offers opportunities for shared treatment strategies. Anti-TNF agents such as adalimumab and infliximab show efficacy in both conditions. For refractory cases, newer drugs, those biologics targeting IL-12/23 and JAK inhibitors targeting the JAK-STAT pathway, are promising but require further validation by new studies. The gut–skin axis further underscores shared pathogenesis. Dysbiosis, characterized by altered gut microbial populations, may influence systemic inflammation detected in both HS and IBD. In conclusion, the co-occurrence of HS and IBD underscores the need for interdisciplinary collaboration between dermatologists and gastroenterologists. Early recognition and integrated care approaches are essential for optimizing treatment and preventing complications. Future research should focus on elucidating the precise mechanisms linking gut and skin inflammation, refining diagnostic tools, and expanding effective therapeutic options for this complex patient population.

## Figures and Tables

**Table 1:** Important original studies investigating the potential bidirectional association between HS and IBD.

Author (Year) (Ref.)	Type of Study (Number of Participants)	Key Findings	Results
Van Der Zee et al. (2012) [19]	Pilot study (158 patients with IBD)	First study investigating the prevalence of HS in patients with IBD	17% in CD patients and 14% in UC patients
Yadav et al. (2016) [20]	Population-based cohort study (679 patients with IBD)	Long-term follow-up on HS incidence in patients with IBD	- 1.18% developed HS over 19.8 years - Strong female predominance
Hung et al. (2021) [14]	Population-based cohort study (2847 patients with IBD and 14,235 matched controls)	HS developed after IBD diagnosis	- IBD was associated with the subsequent development of HS (HR = 2.48; 95%CI, 1.03–5.97)
Killasli et al. (2020) [21]	Cross-sectional study (13,538 HS patients)	Prevalence of IBD in HS patients	Comorbidity for IBD was 3%
Deckers et al. (2017) [22]	Multicenter cross-sectional study (1076 HS patients)	Prevalence of IBD in HS	3.3% (CD = 2.5%, UC = 0.8%)
Egeberg et al. (2017) [13]	Observational cohort study (7732 patients with HS and 4354,137 subjects from the general population)	Risk of IBD in HS	The prevalence (HS vs. general population) was 0.8% and 0.3% (odds ratio 2.04; 1.59–2.62) for CD and 1.3% and 0.7% (odds ratio 1.75; 1.44–2.13) for UC, while the risk of new-onset CD (hazard ratio 2.19; 1.44–3.34) and UC (hazard ratio 1.63; 1.18–2.27) was significantly increased among patients with HS
Shalom et al. (2016) [27]	Cross-sectional study (3207 HS patients and 6412 age- and sex-matched control subjects without HS)	Association of HS with IBD	HS was significantly associated with CD (odds ratio = 2.03, *p* = 0.01) but not with UC (odds ratio = 1.82, *p* = 0.15)
Schneeweiss et al. (2022) [28]	Large national cohort study (6806 patients with HS and 2.376.120 comparator patients without a chronic inflammatory skin disease)	Risk of IBD in HS vs. controls	Increased risk of both UC (HR = 2.30) and CD (CD = 2.70)
Bao et al. (2023) [29]	Mendelian randomization study	First study investigating the causal association between HS and IBD	IBD and its subtypes may have a causal effect on HS, whereas HS does not affect IBD

**Table 2:** Hurley classification for HS.

**Grade 1**	Abscess, single or multiple, without sinus tracts and cicatrization
**Grade 2**	Recurrent abscess with tract formation, single or multiple widely separated lesions
**Grade 3**	Diffuse or almost diffuse involvement, or multiple interconnected sinus tracts and abscesses across the entire area

**Table 3:** Important original studies investigating the characteristics of patients with both HS and CD.

Author (Year) (Ref.)	Type of Study (Number of Participants)	Key Findings	Results
Kamal et al. (2016) [41]	Retrospective study (15 patients with CD + HS)	High rates of perianal and colonic involvement as well as severe HS stages in patients with both HS and CD	- 47% colonic CD, 53% ileocolonic, 67% perianal disease - 93% had Hurley stage II/III
Dumont et al. (2020) [38]	Retrospective case–control study (4645 patients with CD)	More active, severe CD, more severe HS, increased surgical need in patients with coexisting HS and CD compared with patients with CD and without HS	- HS prevalence among patients with CD was 0.95% (44 cases) - 80% of patients with both HS and CD had Hurley II/III HS - Active CD in 56% vs. 40% (*p* < 0.001) - Permanent stoma in 16.8% vs. 2.5% (*p* = 0.002)
Lukach et al. (2018) [40]	Case–control study (38 patients with HS and IBD were identified and matched on age, gender, and IBD type to 136 controls with IBD)	CD + HS patients exhibit more severe colonic involvement and perianal disease compared with patients exhibiting only CD	Patients with HS and CD were significantly more likely to have ileocolonic and perianal CD than patients with CD only (OR 8.31, 95% CI 2.90–23.80 and OR 2.85, 95% CI 1.19–6.81, respectively, *p* < 0.01 for both)
Tandon et al. (2021) [42]	Retrospective case–control study (29 cases of HS, 19 CD and 10 UC, and 145 controls)	HS and CD were more likely to present active perianal disease compared with patients presenting only CD	OR 21.1, 95% CI 6.2 to 71.9, *p* < 0.005

**Table 4:** Major differences between perianal HS and CD.

Category	Perianal HS	Perianal CD
**Extent of Fistulas**	- Sinus tracts and tunnels often confined to superficial/subcutaneous layers - Rarely involve sphincter muscles - Do not extend to the dentate line	- Fistulas often extend to and may involve perianal structures, possibly sphincter muscles - Frequently reach the dentate line
**Clinical Symptoms**	- Painful nodules, abscesses, and purulent draining tunnels - Common also in axillae, groin - Often bilateral - No gastrointestinal symptoms unless comorbid with CD	- Gastrointestinal symptoms: diarrhea, abdominal pain, weight loss - Perianal lesions may also include ulcers, fissures, fistulas
**Laboratory Findings**	- Less frequent and milder anemia than CD - Significantly lower monocyte/lymphocyte ratio (MLR), platelet/lymphocyte ratio (PLR) compared with CD	- Higher prevalence of anemia - Elevated systemic inflammatory markers (MLR, PLR)
**Imaging Findings**	- MRI/US shows bilateral sinus tracts not involving the rectal wall - Absence of rectal wall thickening - Sinus tracts connect to hair follicles	- MRI shows linear, often branching fistulous tracts characterized by internal opening in the anus or low rectum and an external opening to the skin surface - Rectal wall thickening is typical
**Histology Findings**	- Foreign body type granulomas are common (near sinus tracts/hair follicles) - Occasionally shows discrete non-caseating epithelioid granulomas, especially in deep dermis or subcutis	- Non-caseating epithelioid granulomas more frequent - Granulomas may be associated with crypt injury or mucosa

**Table 5:** Genetic and immunopathogenic overlap between HS and IBD.

**Shared Genetic Factors** **NOD2 mutations** Linked to CD; rare association in HS (not classic HS, but in PASH syndrome) ***SULT1B1 and SULT1E1* genes** *-SULT1B1* and *SULT1E1* genes are associated with increased risk of HS and IBD co-occurrence *-SULT1E1*, which encodes an estrogen sulfotransferase, is expressed in adipose tissue and has been shown to be co-expressed with TNF-α ***ELOVL7* gene** Potential protective role against HS and IBD coexistence **Familial Link** HS in first-degree relatives of CD patients suggests genetic susceptibility
**Microbiome Dysregulation** **Dysbiosis** ↑ *E. coli*, ↓ *F. prausnitzii* in patients with HS and IBD **Gut-Skin Axis** Suggests that gut microbiota may influence skin inflammation **TLRs and Inflammasomes** Triggered by microbial components in skin and gut, promoting inflammation **Tobacco Smoke** Activates aryl hydrocarbon receptor and affects microbiota, promotes TNF-α, neutrophil chemotaxis, and follicular plugging
**Immune Dysregulation** **TNF-α** Central pro-inflammatory cytokine in both diseases **IL-1, IL-6, IL-12, IL-17, IL-23** Key cytokines linking HS and IBD **IL-10** Anti-inflammatory cytokine; possibly decreased in both HS and IBD **IL-36 and IL-38** Recently implicated in autoinflammatory diseases including HS and IBD **Th17/Th1 cells** Support epithelial and neutrophilic inflammation **CD161+ T cells** Found in both CD fistulas and HS lesions **Elevated STAT1 signaling in JAK-STAT pathway** Elevated STAT1 signaling is observed in lamina propria T-cells of CD patients (but not in UC), while increased STAT1 mRNA expression is also found in keratinocytes of HS lesions, suggesting STAT1 as a potential molecular link between CD and HS.

**Table 6:** Potential common biologic agents and JAK inhibitors being considered as effective treatment options in patients with coexisting HS and IBD.

Biologic or JAK Inhibitor	HS	CD	UC
Adalimumab	FDA-Approved	FDA-Approved	FDA-Approved
Infliximab	Off-Label use	FDA-approved	FDA-approved
Ustekinumab	Off-Label use	FDA-approved	FDA-approved
Tofacitinib	Off-Label use	not FDA-approved	FDA-approved
Upadacitinib	Off-Label use	FDA-approved	FDA-approved
Guselkumab	Off-Label use	Off-Label use	Off-Label use

## Data Availability

The data described in this study are available upon request from the corresponding author.
